# Rapid Detection of Rifampicin and Isoniazid Resistant *Mycobacterium tuberculosis* Using Genotype MTBDR*plus* Assay in Nepal

**DOI:** 10.1155/2014/648294

**Published:** 2014-10-28

**Authors:** Bijay Kumar Sharma, Shiva Bhandari, Bhagwan Maharjan, Bhawana Shrestha, Megha Raj Banjara

**Affiliations:** ^1^Central Department of Microbiology, Tribhuvan University, Kathmandu, Nepal; ^2^German Nepal Tuberculosis Project (GENETUP), Kathmandu, Nepal

## Abstract

Rapid line probe assay (LPA) can be a practical and rapid alternative to the slow conventional phenotypic drug susceptibility testing (DST) for detection of drug resistant tuberculosis (TB). The purpose of this study is to determine the diagnostic accuracy of Genotype MTBDR*plus*, LPA for TB, and compare its performance with conventional DST. A total of 54 culture samples were analyzed for DST using both conventional proportion method and MTBDR*plus*, where conventional DST identified 43 isolates (79.6%) as drug resistant. Among these 43 drug resistant isolates, 30 isolates (69.7%) were found to be multidrug resistant (MDR). Of all observed mutations using MTBDR*plus*, codon 531 of *rpoB* gene and codon 315 of *katG* gene were found to have highest mutational frequency for RIF resistance (64.7%) and INH resistance (96.8%), respectively. In the present study, MTBDR*plus* assay was shown to have excellent specificity (100%) for both RIF and INH resistance while sensitivity of the assay was little lower with value of 89.4% for RIF resistance and 91.4% for INH resistance. Therefore, the assay can be a rapid, reliable, and promising molecular test for early detection of MDR-TB in Nepal.

## 1. Introduction

Tuberculosis (TB) has been reported from all parts of the world; however, over 95% of cases and deaths due to TB occur in developing countries [1]. MDR-TB rate of 2.6% among new cases and 17.6% among retreatment cases was reported at the latest national survey in Nepal [2]. The emergence of MDR-TB is widely considered to be serious threat to global TB control [3, 4], and in Nepal, being geographically situated between China and India which carry almost 50% of world's MDR-TB burden [5], drug resistance TB is emerging as a national problem.

Random mutation rate of 3 × 10^−7^ to 1 × 10^−9^ per organism per generation is natural for first-line antituberculosis drugs against TB that gives drug resistance [6]. This small proportion of naturally occurring drug resistant mutants, however, rapidly multiplies due to activities like inaccurate or incomplete chemotherapy. While resistance to INH is mainly associated with mutations in the* katG*,* inhA*, and* ahpC* genes [7], resistance to RIF is predominantly linked to mutations in the* rpoB* gene [8].

Processing of sputum specimen followed by culture and drug susceptibility testing (DST) is essential to diagnose drug resistance [9]. Conventional DST is, however, time consuming and there are numerous problems associated with the standardization of tests and the stability of the drugs in different culture media [10]. The slow diagnosis of drug resistance can be a major contributor for the transmission of MDR-TB. Hence, effective control of drug-resistant TB relies on rapid diagnostic assays. Genotype MTBDR*plus* LPA (line probe assay) has been recommended for rapid detection of drug resistant TB by World Health Organization (WHO) [11]. Hence, we conducted this study to evaluate the performance of Genotype MTBDR*plus* in diagnosis of drug resistant TB.

Line probe assay Genotype MTBDR*plus* assay is validated for both direct use on smear-positive pulmonary specimens and on isolates of* Mycobacterium tuberculosis* grown on liquid medium or in solid medium [11]. The assay is based on multiplex polymerase chain reaction (PCR) combined with reverse hybridization on nitrocellulose strips targeting common mutations for RIF and INH resistance. The assay has an additional advantage over other line probe assays because the Genotype MTBDR*plus* assay identifies mutations in the* rpoB* gene (coding for the *β*-subunit of the RNA polymerase) for detection of RIF resistance, mutations in the* katG* gene (coding for the catalase peroxidase) for high-level INH resistance, and mutations in the promoter region of* inhA* gene (coding for the NADH enoyl ACP reductase) for low-levels INH resistance.

Therefore, this study was aimed at determining the patterns of mutations in* rpoB* gene (for detecting RIF resistance) and* katG* and* inhA* genes (for detecting INH resistance) in* Mycobacterium tuberculosis* strains isolated from Nepalese patients and evaluating the performance of Genotype MTBDR*plus* against conventional DST.

## 2. Materials and Methods

### 2.1. Participants

This study was conducted in German Nepal Tuberculosis Project (GENETUP), national reference laboratory for TB drug sensitivity testing, Kathmandu, Nepal. Sixty-two sputum samples were processed in GENETUP and all these samples were referred from 9 directly observed treatment short-course (DOTS) plus treatment centre where the patients were taking their treatment. These treatment centres are located in five development regions of Nepal.

### 2.2. Test Methods

Specimens were obtained in sterile, leak proof, wide mouth, transparent, and stopper plastic containers. Fluorescence microscopy of the collected isolates was performed and all acid fast positive samples were cultured on Lowenstein-Jensen (LJ) medium. Culture positive samples were assessed for drug resistance using phenotypic conventional DST and molecular genotypic assay where the former is considered as gold standard for evaluation purpose.

Proportion method was used on LJ medium for DST at critical concentrations of 0.2 *μ*g/mL for INH and 40 *μ*g/mL for RIF. Final reading of the test was done after 6 weeks of incubation at 37°C and the strains were considered resistant if the proportion of resistant bacteria was higher than 1%. Genotype MTBDR*plus* assay was also performed on mycobacterial cultures according to manufacturer's (Hain Lifescience) instructions. Briefly, genomic DNA was extracted from bacterial culture by suspending few colonies in 300 *μ*L of molecular biology grade water and incubated for 20 minutes at 95°C in water bath and further 15 minutes in an ultrasonic bath with final spinning for 5 minutes at a speed of 12000 rpm. Polymerase chain reaction (PCR) was performed using primers and deoxyribonucleotide precursors provided by manufacturer and subsequent hybridization was done using the Twin-Cubator (Hain Lifescience) according to manufacturer's recommendations. Hybridized amplicons were colorimetrically detected using streptavidin-conjugated with alkaline phosphatase and substrate buffer. Finally, strip containing hybridized amplicons were air dried and fixed on evaluation paper for interpretation of drug resistance patterns of the isolates. During interpretation, an isolate was considered sensitive when all wild type probes produce band but no such bands in mutation probes. Missing of band development in any of the wild types probes or band development in any of the mutation probes suggests resistant type of isolates. Strips which tested positive for probes of amplification control, conjugate control, and locus control of targeted gene were only interpreted which otherwise are considered invalid.

### 2.3. Statistical Methods

The study was conducted and reported in compliance with the STARD guidelines. SPSS software 20.0 was used for data analysis. Percentages and mean were used for comparing the measures of diagnostic accuracy while http://vassarstats.net/clin1.htm/ was used to determine sensitivity, specificity and positive and negative predictive value in 95% confidence interval.

## 3. Results

### 3.1. Participants

This study was conducted from September 2013 to March 2014. Both female (45.1%) and male (54.9%) TB patients' sputum specimens were included in the study. The age range of patients whose sputum specimen were collected ranges from 17 to 72 with mean age of 32. Among the selected DOTS plus centre located in different part of Nepal, sample size of 11.2%, 14.5%, 41.9%, 17.7%, and 14.5% was chosen from eastern, central, western, mid-western, and far-western part of the country, respectively (Table 1). This sample size was based upon the total number of TB patients attending the treatment centres. All the cases chosen were pulmonary TB patient with symptoms of chest pain, night fever, weight loss, blood mixed sputum, and so forth. Only those sputum specimens that were diagnosed sputum smear positive in the treatment centre only included in the study while sputum smear negative samples were excluded from the study. Referred samples were selected from the treatment sites on the basis of nonprobability based convenience sampling. Patient's data were collected on the basis of case record forms obtained from treatment centres.

### 3.2. Test Results

**Figure 1:**
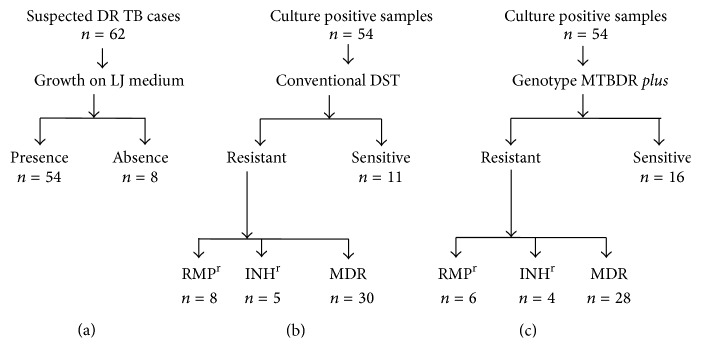
Flow diagram of positive TB cultures (a) and their drug sensitivity pattern in conventional DST (b) and GenoType MTBDR*plus* (c).

All 62 samples collected for the study were acid fast positive by fluorescence microscopy. Out of these 62 samples examined, 54 samples were culture positive in LJ medium and the remaining 8 samples do not show any growth. Hence, only 54 samples were processed for both conventional and genotypic drug susceptibility testing (Figure 1). LJ proportion DST method identified 11.1%, 7.4%, 51.8%, and 29.6% of these 54 strains as RIF monoresistant, INH monoresistant, MDR, and sensitive strains, respectively, while using Genotype MTBDR*plus* assay, the corresponding percentage was 14.8%, 9.2%, 55.5%, and 20.3%, respectively (Table 2).

### 3.3. Estimates

Considering conventional DST as gold standard test sensitivity, specificity, positive predictive value, and negative predictive value of the assay for RIF resistance were found to be 89.4% (95% CI, 74.2%–96.5%), 100% (95% CI, 75.9%–100%), 100% (95% CI, 87.3%–100%), and 80% (95% CI, 87.3%–100%), respectively (Table 3), while the corresponding percentage for INH resistance was found to be 91.4% (95% CI, 75.8%–97.7%), 100% (95% CI, 79%–100%), 100% (95% CI, 86.6%–100%), and 86.3% (95% CI, 64%–96.4%), respectively (Table 4).

Among 34 all rifampicin drug resistant isolates (including both RIF monoresistant and MDR isolates) identified by MTBDR*plus* assay, 23.5% strains (8/34) (Table 5) had point mutation and 76.4% strains (26/34) had multiple mutation (Table 6) in* rpoB* gene. Similarly, among 32 all isoniazid drug resistant isolates (including both INH monoresistant and MDR isolates) identified by MTBDR*plus* assay, 3.1% strains (1/32) had point mutation and 93.7% strains (30/32) had multiple mutations in* katG* gene (Table 5) and 3.1% strains (1/32) had multiple mutation in* inh*A gene (Table 6).

Among the resistant isolates studied, 28 strains were reported to be MDR by Genotype MTBDR*plus* assay. Mutation pattern of these isolates was found to be varied with altogether 10 different patterns of mutation. Most prominent mutation of MDR isolates was observed in the 531 gene region of* rpoB* gene, that is, S531L mutation (resistance due to* katG* MUT3 probe) (16/28; 57.1%) for RIF resistance and 315 region of* katG* gene for INH resistance (27/28; 96.4%) (Table 7).

## 4. Discussion

The present study shows that Genotype MTBDR*plus* assay has excellent specificity (100%) for detection of RIF and INH resistance TB in culture isolates while sensitivity of the assay has some lesser accuracy for detection of RIF (89.4%) and INH (91.4%) resistance. This suggests that conventional DST should always be carried out before making conclusive test result even though Genotype MTBDR*plus* assay has advantage of rapid turnaround time. In our study, most Nepalese culture isolates identified by conventional DST were reported to be drug resistant (43/54, 79.6%) and among those drug resistant TB isolates most isolates were multidrug resistant (30/43, 69.7%) compared to monoresistant (13/43, 30.2%). Therefore, Genotype MTBDR*plus* assay can be significantly valuable for TB patient management and control of transmission of drug resistant TB in Nepal.

In our study, discordance of sensitivity of Genotype MTBDR*plus* with phenotypic DST was reported. Sensitivity of the assay for RIF resistance in this study was lower than reported from various meta-analyses, where calculated pooled sensitivities of assay range from 98.1% (95% CI, 95.9%–99.1%) [12] to 99% (95% CI, 96%–100%) [13] while it was higher than in South Africa (85.7%, 95% CI, 57.2–98.2%) [14]. Similarly, sensitivity of the assay for INH resistance in this study was higher than reported in Caribbean (35–73%) [15], Germany (88.4%) [7], and South Africa 62.1% [14] while it was lesser than in Nepal [16]. Comparing to various meta-analyses studies where calculated pooled sensitivities of assay range from 84.3% (95% CI, 76.6%–89.8%) [12] to 96% (93–98%) [13], sensitivity of the assay in our study for INH resistance is moderate. Specificity of the assay for both RMP resistance 100% (95% CI, 77.1%–100%) and INH resistance 100% (95% CI, 79.9%–100%) was excellent in our study as obtained in other meta-analyses where very close data of 99% [13] to 99.5% [12] was reported. Discordance of sensitivity of Genotype MTBDR*plus* with phenotypic DST may be due to mutations in other gene regions which are not targeted by the assay like* ahp*C gene encoding alkyl hydroperoxide reductase for INH resistance [17].

Among various mutations observed using Genotype MTBDR*plus* assay, majority of the drug resistant isolates had multiple mutations (26/34 i.e., 76.4% for RIF resistance, 31/32 i.e., 96.8% for RIF resistance) in our study. These multiple mutations are believed to be more probable in high TB incidence places, which is true in our study as 45% of Nepalese people are infected with TB [2]. All RIF resistant isolates in this study were reported to have their mutations in 81-bp core rifampicin resistance determining region (RRDR) of* rpoB* codons 507 to 533. This is in agreement with a study done in Belgium [18] and Nepal [19] where over 96% of RIF resistant* M. tuberculosis* strains were reported to have mutations in RRDR of* rpoB* region.

In case of RIF resistance isolates, missense mutation at codons 531 and 516 of* rpoB* gene was observed. S531L missense mutation that led amino acid substitutions of serine to threonine was most common in* rpoB* gene accounting for 50% (17/34) of all RIF resistant isolates. Similar result was found in South Vietnam [20] but it was found to be less frequent than in Thailand (63.6%) [21] and India (72%) [22] and more frequent than in a study from Nepal (37.14%) [16]. Amino acid substitution of aspartate to valine at 516 codon of* rpoB* gene was observed in 23.5% (8/34) of the RIF resistant strains studied. This frequency was higher than recent studies reported from Nepal (5.71%) [16] and India (3.03%) [22]. Although various frequencies of mutation (2.8%–40%) are observed geographically in different regions of the world at codon 526 of* rpoB* gene substituting amino acid histidine [16, 19, 23, 24], no mutation was observed in this gene region in this study. In the present study, most point mutations were observed at codons 531 and 516. This result coincides with several other studies that had shown codons 516 or 531 being the most common region with point mutations [6, 19, 25].

Of all INH resistant strains, 93.7% (30/32) of strains have S315T mutation in* katG* region that led amino acid serine substitution to threonine. This is similar to the result found in Thailand where 90% of all isoniazid resistance isolates had mutation in the* katG* gene [21], but this was higher than other studies done in Nepal [16, 19, 26]. In this study, only 3.1% of the INH resistant isolates were observed to have mutation in the promoter region of* inhA* region that led amino acid substitution of cysteine to threonine and this frequency was lower than recent studies done in Nepal where around 13% of INH resistant isolates had mutation in* inhA* gene region [16, 19]. High level and low level of INH resistance were shown to be associated with codon 315 of* katG* gene (50–90%) and regulatory region of* inhA* gene (20–35%), respectively, by various studies [6, 27] which coincides with our study for* katG* mutation but not for* inhA* mutation. However, studies from many countries have confirmed variability in the contribution of different mutations to INH resistance [14, 19, 21]; hence, contribution of* inhA* mutation (3.1%) for INH resistance may be low in our setting.

In our study, Genotype MTBDR*plus* assay has shown excellent specificity and high sensitivity for detection of rifampicin and isoniazid resistant TB when used on culture isolates. Hence, in a setting like Nepal where there is high TB prevalence rate, Genotype MTBDR*plus* assay can be effectively used for rapid screening of drug resistant TB, and for improved sensitivity, additional probes can be integrated in the assay.

## 5. Conclusion

As numbers of drug resistant MTB isolates were reported to be very high among study population in our study, it is significantly important not to miss any isolate during diagnosis so that the worst form of TB (e.g., multidrug resistant TB, extensively drug resistant TB, or pan resistant TB) can be prevented timely. As Genotype MTBDR*plus* is a PCR based technique, this technique can easily detect very low level of resistant bacteria, therefore, giving very less chance to miss any TB isolates. Hence, Genotype MTBDR*plus* can be a promising tool for TB diagnosis, treatment guidance, and surveillance in resource-limited nations like Nepal.

## Figures and Tables

**Table 1:** Demographic characteristics of the TB cases.

Characteristics	Value
Female sex—number (%)	28 (45.1)
Male sex—number (%)	34 (54.9)
Age—yr	
Mean age	32
Range	17–72
Geographic region—number (%)	62 (100)
Eastern	7 (11.2)
Central	9 (14.5)
Western	26 (41.9)
Mid-Western	11 (17.7)
Far-Western	9 (14.5)
Acid fast positive samples—number (%)	62 (100)
Pulmonary TB cases—number (%)	62 (100)

**Table 2:** Conventional phenotypic DST for detection of RIF and INH resistance.

Susceptibility	Number of isolates (*n* = 54)	
	Genotype MTBDR*plus* (%)	LJ proportion DST (%)
RIF monoresistant	6 (11.1)	8 (14.8)
INH monoresistant	4 (7.4)	5 (9.2)
MDR-TB (resistant to RIF and INH)	28 (51.8)	30 (55.5)
Susceptible to RIF and INH	16 (29.6)	11 (20.3)

^*^DST: drug susceptibility testing; RIF: rifampicin; INH: isoniazid.

**Table 3:** Comparison of Genotype MTBDR*plus* and phenotypic DST for RIF resistance.

Genotype MTBDR*plus *	LJ proportion DST method		Total
	RIF resistant	RIF sensitive	
RIF resistant	34	0	34
RIF sensitive	4	16	20
Total	**38**	**16**	**54**

**Table 4:** Comparison of Genotype MTBDR*plus* and phenotypic DST for INH resistance.

Genotype MTBDR*plus *	LJ proportion DST method		Total
	INH resistant	INH sensitive	
INH resistant	32	0	32
INH sensitive	3	19	22
Total	**35**	**19**	**54**

**Table 5:** Point mutations observed using Genotype MTBDR*plus* assay.

LPA probes	Mutation site	Mutation detected	Number of isolates (%)
*rpoB *			All RIF^r^ isolates (*n* = 34)
MUT 1	516	D516V	2 (5.8)
MUT 3	531	S531L	1 (1.9)
WT 7	526–529		3 (8.8)
WT 8	530–533		2 (5.8)
*katG *			All INH^r^ isolates (*n* = 32)
WT	315		1 (3.1)

^*^r: resistant.

**Table 6:** Multiple mutations observed using Genotype MTBDR*plus* assay.

LPA probes	Mutation sites	Mutation detected	Number of isolates (%)
*rpoB *			All RIF^r^isolates (*n* = 34)
WT 3, 4, MUT1	513–518	D516V	5 (14.7)
WT8, MUT 3	530–533	S531L	16 (47.1)
WT 8, MUT1	531, 533	D516V	1 (1.9)
WT 1, 6, 7, 8	505–509, 518–533		1 (1.9)
WT 6, 7, 8	518–533		1 (1.9)
WT 3, 4	513–518		1 (1.9)
WT 2, 3	510–516		1 (1.9)
*katG *			All INH^r^isolates (*n* = 32)
WT, MUT1	315	315 (S315T1)	29 (90.6)
WT, MUT 2	315	315 (S315T2)	1 (3.1)
*inhA *			All INH^r^isolates (*n* = 32)
WT1, MUT1	−15, −16	C15T	1 (3.1)

**Table 7:** Pattern of multiple gene mutations observed in MDR TB isolates using Genotype MTBDR*plus* assay.

Pattern of gene mutations in MDR-TB strains	Number of isolates (%) (*n* = 28)
*rpoB *MUT3, *rpoB *WT8,* katG *MUT1,* katG *WT	14 (50)
*rpoB *MUT3, *rpoB *WT8,* katG *WT	1 (3.5)
*rpoB *WT8,* katG *MUT1,* katG *WT	2 (7.1)
*rpoB *MUT3,* inhA *MUT1, *inhA *WT1	1 (3.5)
*rpoB *WT7,* katG *WT	1 (3.5)
*rpoB *WT2,* rpoB *WT3,* katG *MUT1,* katG *WT	1 (3.5)
*rpoB *WT3,* rpoB *WT4,* katG *MUT1,* katG *WT	1 (3.5)
*rpoB *MUT1,* rpoB *WT3,* rpoB *WT4,* katG *MUT1,* katG *WT	5 (17.8)
*rpoB *MUT1, *katG *MUT2,* katG *WT	1 (3.5)
*rpoB *WT7,* katG *MUT1,* katG *WT	1 (3.5)

^*^MDR: multi-drug resistant.

## References

[1] World Health Organization (2012). *Global Tuberculosis Report*.

[2] National Tuberculosis Centre (2012). National Tuberculosis Programme of Nepal. *Annual Report*.

[3] Migliori G. B., Loddenkemper R., Blasi F., Raviglione M. C. (2007). 125 years after Robert Koch's discovery of the tubercle bacillus: the new XDR-TB threat. Is “science” enough to tackle the epidemic?. *European Respiratory Journal*.

[4] Raviglione M. C., Smith I. M. (2007). XDR tuberculosis: implications for global public health. *New England Journal of Medicine*.

[5] Wright A., Zignol M., Van Deun A. (2009). Epidemiology of antituberculosis drug resistance 2002-07: an updated analysis of the Global Project on Anti-Tuberculosis Drug Resistance Surveillance. *The Lancet*.

[6] Gillespie S. H. (2002). Evolution of drug resistance in *Mycobacterium tuberculosis*: clinical and molecular perspective. *Antimicrobial Agents and Chemotherapy*.

[7] Hillemann D., Weizenegger M., Kubica T., Richter E., Niemann S. (2005). Use of the genotype MTBDR assay for rapid detection of rifampin and isoniazid resistance in *Mycobacterium tuberculosis* complex isolates. *Journal of Clinical Microbiology*.

[8] Hillemann D., Rüsch-Gerdes S., Richter E. (2007). Evaluation of the GenoType MTBDRplus assay for rifampin and isoniazid susceptibility testing of *Mycobacterium tuberculosis* strains and clinical specimens. *Journal of Clinical Microbiology*.

[9] Varma J. K., Wiriyakitjar D., Nateniyom S. (2007). Evaluating the potential impact of the new Global Plan to Stop TB: Thailand, 2004-2005. *Bulletin of the World Health Organization*.

[10] Victor T. C., Warren R., Butt J. L. (1997). Genome and MIC stability in *Mycobacterium tuberculosis* and indications for continuation of use of isoniazid in multidrug-resistant tuberculosis. *Journal of Medical Microbiology*.

[11] World Health Organization (2008). *Molecular Line Probe Assays for Rapid Screening of Patients at Risk of Multidrug-Resistant Tuberculosis (MDR-TB): Policy Statement*.

[12] Ling D. I., Zwerling A. A., Pai M. (2008). GenoType MTBDR assays for the diagnosis of multidrug-resistant tuberculosis: a meta-analysis. *European Respiratory Journal*.

[13] Bwanga F., Hoffner S., Haile M., Joloba M. L. (2009). Direct susceptibility testing for multi drug resistant tuberculosis: a meta-analysis. *BMC Infectious Diseases*.

[14] Dorman S. E., Chihot V. N., Lewis J. J. (2012). Genotype MTBDRplus for direct detection of mycobacterium tuberculosis and drug resistance in strains from gold miners in South Africa. *Journal of Clinical Microbiology*.

[15] Akpaka P. E., Baboolal S., Clarke D., Francis L., Rastogi N. (2008). Evaluation of methods for rapid detection of resistance to isoniazid and rifampin in *Mycobacterium tuberculosis* isolates collected in the Caribbean. *Journal of Clinical Microbiology*.

[16] Dahal B., Adhikari N., Shah Y., Simkhada R., Maharjan B., Shrestha B. (2013). Evaluation of Genotype MTBDRplus Assay for identifying Multidrug Resistant Mycobacterium tuberculosis isolates in Nepal. *Janaki Medical College Journal of Medical Science*.

[17] Ma X., Wang H., Deng Y. (2006). rpoB gene mutations and molecular characterization of rifampin-resistant *Mycobacterium tuberculosis* isolates from Shandong Province, China. *Journal of Clinical Microbiology*.

[18] Traore H., Fissette K., Bastian I., Devleeschouwer M., Portaels F. (2000). Detection of rifampicin resistance in *Mycobacterium tuberculosis* isolates from diverse countries by a commercial line probe assay as an initial indicator of multidrug resistance. *International Journal of Tuberculosis and Lung Disease*.

[19] Poudel A., Nakajima C., Fukushima Y. (2012). Molecular characterization of multidrug-resistant *Mycobacterium tuberculosis* isolated in Nepal. *Antimicrobial Agents and Chemotherapy*.

[20] Huyen M. N. T., Tiemersma E. W., Lan N. T. N. (2010). Validation of the GenoType MTBDRplus assay for diagnosis of multidrug resistant tuberculosis in South Vietnam. *BMC Infectious Diseases*.

[21] Rienthong D., Rienthong S., Boonin C., Woraswad S., Kasetjaroen Y. (2009). Rapid detection for early appearance of Rifampin and Isoniazid resistance in *Mycobacterium tuberculosis*. *Siriraj Medical Journal*.

[22] Yadav R. N., Singh B. K., Sharma S. K. (2013). Comparative evaluation of genotype MTBDRplus line probe assay with solid culture method in early diagnosis of multidrug resistant tuberculosis (MDR-TB) at a tertiary care centre in India. *PLoS ONE*.

[23] Yue J., Shi W., Xie J., Li Y., Zeng E., Wang H. (2003). Mutations in the rpoB gene of multidrug-resistant *Mycobacterium tuberculosis* isolates from China. *Journal of Clinical Microbiology*.

[24] Raveendran R., Wattal C., Oberoi J. K., Goel N., Datta S., Prasad K. J. (2012). Utility of GenoType MTBDRplus assay in rapid diagnosis of multidrug resistant tuberculosis at a tertiary care centre in India. *Indian Journal of Medical Microbiology*.

[25] Drobniewski F. A., Wilson S. M. (1998). The rapid diagnosis of isoniazid and rifampicin resistance in *Mycobacterium tuberculosis*—a molecular story. *Journal of Medical Microbiology*.

[26] Marahatta S. B., Gautam S., Dhital S. (2011). KatG (SER 315 THR) gene mutation in isoniazid resistant Mycobacterium tuberculosis. *Kathmandu University Medical Journal*.

[27] Miotto P., Piana F., Cirillo D. M., Migliori G. B. (2008). Genotype MTBDRplus: a further step toward rapid identification of drug-resistant *Mycobacterium tuberculosis*. *Journal of Clinical Microbiology*.

